# Rapid Cure of Lichen Ruber by Hypodermic Injections of Arsenic

**Published:** 1882-02

**Authors:** 


### Rapid Cure of Lichen Ruber by Hypodermic Injections of Arsenic.

Köbner reports the cure of what seems to be a well-marked case of lichen ruber, by means of hypodermic injections of Fowler's solution. The eruption was more or less general. That the rapidity of cure was due to the hypodermic method is proven by the facts that the case had already been ordinarily treated, but without benefit; and also once during treatment the patient rebelled against the plan, and the solution was then given by the mouth, but improvement failed to continue. The injections were then renewed, and a rapid recovery followed. The intense itching present yielded to the first few injections. The injections were made at intervals of one or two days, and in all but one fluid drachm of the solution, each injection averaging about four minims of the solution with two parts of water. Six months afterward there had been no reappearance of the eruption.—*Deutsche Med. Wochenschr.*

